# Heat Shock Protein 27 Levels Predict Myocardial Inhomogeneities in Hemodialysis Patients

**DOI:** 10.1155/2022/5618867

**Published:** 2022-05-19

**Authors:** Andrzej Jaroszyński, Todd T. Schlegel, Jerzy Mosiewicz, Renata Stępień, Wojciech Dąbrowski

**Affiliations:** ^1^Collegium Medicum, Jan Kochanowski University of Kielce, Kielce, Poland; ^2^Department of Molecular Medicine and Surgery, Karolinska Institute, Stockholm, Sweden; ^3^Nicollier-Schlegel SARL, 1270 Trélex, Switzerland; ^4^Department of Internal Medicine, Medical University of Lublin, Lublin, Poland; ^5^Department of Anaesthesiology and Intensive Therapy, Medical University of Lublin, 20-954 Lublin, Poland

## Abstract

**Background:**

Sudden cardiac death (SCD) is the single major cause of death in hemodialysis (HD) patients. QRS-T angle is an established marker of global repolarization heterogeneity associated with electrical instability and SCD. Heat shock protein 27 (HSP27) plays an important, protective role against noxious factors in the cardiovascular (CV) system. This study is aimed at assessing whether low HSP27 is associated with myocardial inhomogeneities in HD patients, as expressed by increases in the spatial QRS-T angle.

**Methods:**

Clinical data and biochemical, echocardiographic, and electrocardiographic parameters were evaluated in 182 HD patients. Patients were split into normal and abnormal QRS-T angle groups.

**Results:**

Patients with abnormally high QRS-T angles were older and had higher prevalence of diabetes as well as myocardial infarction, higher left ventricular mass index (LVMI) and C-reactive protein, worse oxidant/antioxidant status, and lower ejection fraction and HSP27. Multiple regression analysis revealed that abnormal QRS-T values were independently, negatively associated with serum HSP27 and positively associated with LVMI.

**Conclusions:**

Low HSP27 levels are associated with increased heterogeneity of myocardial action potential, as expressed by increased spatial QRS-T angle.

## 1. Introduction

Renal registry data shows that sudden cardiac death (SCD) is the single major cause of death in hemodialysis (HD) patients, accounting for approximately 26% of all mortality [1]. The pathogenesis of SCD in HD patients is complex, multifactorial, and not completely understood. Additionally, clear differences in terms of the pathophysiology and cause of SCD exist between HD patients and the general population [1–4].

The QRS-T angle is the spatial angle between the three-dimensional vectorcardiographic representation of QRS complex and T-wave loops (ventricular repolarization and depolarization). The QRS-T angle is an established marker of global repolarization heterogeneity associated with electrical instability and SCD [5–10]. Well-documented evidence exists that QRS-T angle is a sensitive, powerful, and reliable risk stratifier for cardiac events and especially for SCD in the general population and in various clinical settings [5, 6, 11–14], including HD patients [7, 8, 15–19].

Heat shock proteins (HSP) are chaperone proteins that protect cells against noxious factors and interact with other proteins to facilitate normal cellular functions. Heat shock protein 27 (HSP27) is a part of the HSP family playing an important role in cardiovascular (CV) system. HSP27 is both a biomarker of CV disease and a potential therapeutic target [20–23]. Evidence exists that HSP27 functions as an antioxidant, exerts cytoprotective effects, inhibits apoptosis, and attenuates myocardial injury [20, 21, 23–26]. Recent studies suggest that HSP27 acts as a linking molecule influencing CV mortality in HD patients [23, 24].

The purpose of this study was to assess whether low levels of HSP27 in HD patients are associated with heterogeneity of myocardial action potential as expressed by QRS-T angle.

## 2. Materials and Methods

### 2.1. Patients

This analysis was performed using digital ECG recordings collected as part of a recently published study that investigated the role of HSP27 in the prediction of cardiac mortality in HD patients [27]. Patient characteristics, inclusion/exclusion criteria, and protocols for this study have previously been described in details [27]. In brief, 202 HD patients qualified for the study underwent ECG recordings, echocardiography, and blood sampling. The study was performed in accordance with the Declaration of Helsinki and was approved by institutional review board—“Bioethical Committee of Medical University of Lublin” (KE-0254/125/2011).

### 2.2. Biochemical Variables

Biochemical routine tests including electrolytes, hemoglobin, creatinine, urea, phosphates, C-reactive protein (CRP), total protein, albumin, intact parathormone (PTH), lipid profile, and troponin T were measured in all patients by automated analyzers. Both serum total antioxidant capacity (TAC) and total oxidant capacity (TOC) were evaluated by using colorimetric methods (Immundiagnostik AG, Germany), as previously described in detail [27]. Serum HSP27, NT-proBNP, and oxidized LDL (oxLDL) were measured by the ELISA method (Biomedica). All measurements were performed the day after the dialysis session.

### 2.3. Echocardiographic Examination

All echocardiographic measurements were performed by a cardiologist who was blinded to patients' clinical data according to the American Society of Echocardiography recommendations as described previously. LVH was diagnosed when LVMI exceeded 130 g/m^2^ in males or 110 g/m^2^ in females [28].

### 2.4. Electrocardiography Recordings

Surface 12-lead resting ECG was recorded in each patient using a Cardiax device (IMED Co Ltd, Budapest, Hungary). ECGs were recorded in an electrically shielded and noise-proof room with subjects lying in the horizontal position after at least 5 min rest. All ECGs were obtained the day after the dialysis session, when data for all other tests were also obtained. The 10 s recordings were automatically averaged to a single beat and transformed into three orthogonal leads using the inverse Dower method. The projections of the maximum vectors of QRS and T-waves in the frontal, transverse, and left sagittal planes and on the *X*-, *Y*-, and *Z*-axes were then obtained. Next, the spatial QRS-T angle values were automatically calculated from the maximum spatial QRS and T vectors. An abnormal spatial QRS-T angle was defined as a spatial QRS-T angle > 116 degrees for females and >130 degrees for males [29].

### 2.5. Statistical Analysis

Statistical analysis was performed using Statistica Version 10 as described in detail previously [27]. Due to the inability to estimate population size in our previous study [27], no calculation of the sample size was also performed in the present study [27]. All available HD patients from a previous study for whom an ECG was available were included. Linear regression analysis was carried out by using the Pearson or Spearman test, as appropriate. Nonnormally distributed data were transformed logarithmically prior to analysis. For further analysis, patients were divided into normal and abnormal QRS-T groups. Significance of differences between QRS-T angle groups was assessed using a Student *t*-test. In order to estimate the potential influence of various factors on QRS-T, multiple stepwise regression analysis was carried out. The model included parameters that showed differences with *p* < 0.05 between normal and abnormal QRS-T groups. Receiver operating characteristics (ROC) curves were constructed to determine optimal cut-off points for HSP27 in predicting abnormal QRS-T values. Probability values of *p* < 0.05 were accepted as significant.

## 3. Results and Discussion

### 3.1. Results

From 202 HD patients included in the original study, digital ECGs were unavailable or unreadable in 20 patients. The remaining 182 subjects were included in the present study.

The causes of end-stage failure were as follows: diabetes mellitus (*n* = 77), glomerulonephritis (*n* = 38), hypertensive nephropathy (*n* = 18), polycystic kidney disease (*n* = 7), obstructive nephropathy (*n* = 5), chronic pyelonephritis (*n* = 5), and unknown/unsure (*n* = 32).

**Figure 1:**
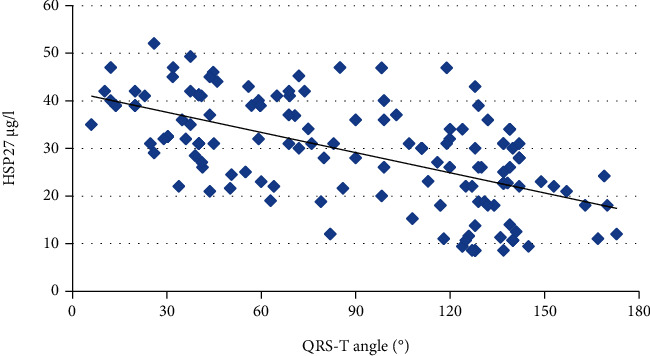
Relation between QRS-T angle and HSP27 levels (*r* = −0.612). HSP27: heat shock protein 27.

The mean ± SD value of the QRS-Tangle in HD patients was 104.8 ± 25.3, with statistically higher values in the abnormal QRS-T group (150.3 ± 12.8) than in the normal QRS-T group (76.9 ± 21.7; *p* < 0.001). Subjects in the abnormal QRS-T group were older (*p* = 0.011) and had higher prevalence of both prior infarction (*p* < 0.001) and diabetes (*p* = 0.001). With regard to echocardiographic parameters, patients with abnormal QRS-T had higher LVMI and lower LVEF than patients with normal QRS-T (*p* < 0.001 in both cases). Regarding biochemical indices, patients with abnormal QRS-T had marginally higher troponin T levels, but significantly higher CRP (*p* = 0.021) as well as TOC and oxLDL levels (*p* < 0.001 in both), and lower TAC levels (*p* = 0.018). The demographic, clinical, and biochemical data of the studied groups are shown in Table 1. Significant relations were found between QRS-T angle and HSP27 (*r* = −0.612, *p* < 0.001) (Figure 1), QRS-T angle and oxLDL (*r* = 0.571, *p* < 0.001), QRS-T angle and TOC (*r* = 0.548, *p* < 0.001), and QRS-T angle and TAC (*r* = −0.489, *p* < 0.001).

The results of multiple regression analysis showed that QRS-Tangle values were independently and inversely associated with HSP-27 levels and independently and positively associated with both TOC and LVMI (Table 2).

**Figure 2:**
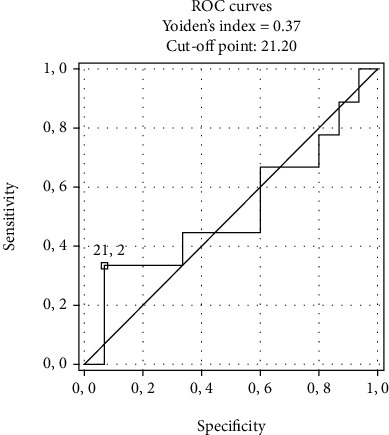
Receiver operating characteristic (ROC) curve of heat shock protein 27 (HSP27) in predicting abnormal QRS-T angle.

The ROC analysis of HSP27 as a predictor of abnormal QRS-T showed an AUC of 0.643 with sensitivity and specificity of 0.645 and 0.611, respectively. The optimal cut-off point for HSP27 was 21.2 *μ*g/L. The ROC curves are presented in Figure 2 and in Table 3.

## 4. Discussion

The key finding of our study is that low serum HSP27 level is an independent and strong predictor of abnormal QRS-T angle in HD patients.

Interest in the QRS-T angle dates back to 1934, when Wilson developed the concept of a “ventricular gradient.” Recently, there has been renewed interest in the QRS-T angle. Abnormalities in depolarization mirror structural abnormalities, while those in repolarization reflect changes in regional action potential duration and the direction of repolarization sequence associated with electrical instability and SCD. QRS-T angle is an established marker of global repolarization heterogeneity, possibly related to underlying structural and functional myocardial abnormalities [3, 6–8, 11, 14–16, 18]. In our study, abnormal QRS-T angle was found in 30% of HD patients. It is in agreement with the results of de Bie et al. [19], who applied a similar methodology and the same cut-off points for normal and abnormal QRS-T values. Some other authors [7, 16] found abnormal QRS-T angle in 40% of HD patients. This difference may be due to methodological differences in QRS-T angle calculation and differences between cut-off values reported in different studies, as well as the differences in the prevalence of comorbidities in the groups of the studied patients. Good evidence exists that in HD patients, widened QRS-T angle predicts both all-cause mortality and CV mortality and is particularly helpful in predicting SCD [7, 16, 18, 19]. Although it is well known that QRS-T angle reflects heterogeneity of the myocardial action potential, the mechanisms linking abnormal repolarization with clinical outcomes remain unclear. In our study, we have shown that low HSP27 and high LVMI were independent predictors of high QRS-T angle. Increased left ventricular mass (LVM) is highly prevalent in HD patients and universally associated with cardiovascular morbidity and mortality [30, 31], including SCD [32]. The relation between QRS-T and LVMI is in line with some but not all previous studies. In the study by Tereshchenko et al. [7], as in our study, the authors found a relationship between widened QRS-T angle and left ventricular hypertrophy, but not with the left ventricular ejection fraction (LVEF). In the study by de Bie et al. [19], contrary to our study, the authors found a relationship between QRS-T angle and the LVEF but not LVMI.

To our knowledge this is the first study demonstrating a relationship between widened QRS-T angle and low HSP27. HSP27 exerts cytoprotective effects, has antioxidant properties, inhibits apoptosis, and participates in protein repair. There is also growing evidence that HSP27 exerts cardioprotective effects [23, 26, 33]. Our results may be in line with our previous study showing that low HSP27 levels are associated with increased cardiac mortality, including SCD [27]. Recent studies have revealed that cardioprotective effects may result from antiapoptotic as well as antioxidant properties. In our study, we found differences in oxidative parameters between normal and abnormal QRS-T groups. Moreover, we found differences in serum oxLDL between normal and abnormal QRS-T groups. While these differences lost their significance in multiple regression analysis, they might nonetheless play a role in myocardial pathology in HD patients. Similarly, our study revealed differences between serum oxLDL between normal and abnormal QRS-T angle groups. Recent studies have shown that HSP27 may contribute to the reduction of LDL oxidative modification, thus demonstrating that HSP27 plays a protective role in atherogenesis [27, 34, 35]. In addition, our previous study showed that inflammation may play a role in the pathogenesis of myocardial inhomogeneities in HD patients [36], and HSP27 is known to be involved in modulating inflammation. It is worth emphasizing that the results of ROC analysis revealed that the HSP27 cut-point value predicting abnormal QRS-T angle was similar to the HSP27 value predicting the occurrence of contrast-induced nephropathy [24]. Given the above results, we can conclude that low HSP27 levels are associated with increased heterogeneity of myocardial action potential as expressed by the QRS-T angle. However, at this stage of research, we cannot determine whether the relationship is merely descriptive or whether the HSP27 protein is involved in the pathogenesis of increased myocardium repolarization heterogeneity. To determine if HSP27 is only a marker of disease severity versus a potential therapeutic target requires further studies.

Moreover, our study also had other limitations. First, we evaluated HSP27 level as well and spatial QRS-T angle values only once in each patient. However, it is likely that serial rather than single measurements of both measures might have better characterized overall results. Second, the inverse Dower transform utilized for derivation of the *X*, *Y*, and *Z* leads by our Cardiax device is not necessarily the scientifically most optimal transform [37].

## 5. Conclusions

Low HSP27 levels are associated with increased heterogeneity of myocardial action potential, as expressed by increased spatial QRS-T angle.

## Figures and Tables

**Table 1:** Basic demographic data and clinical and biochemical data of patients.

Parameter	All patients *n* = 182	Abnormal QRS-T *n* = 54	Normal QRS-T *n* = 128	*p*
Age (years)	70.8 ± 7.64	73.1 ± 7.78	69.1 ± 7.76	0.011
HD vintage (months)	58.04 ± 25.89	59.32 ± 25.12	57.61 ± 25.77	0.406
MI (%)	31.9	44.4	26.6	<0.001
Diabetes mellitus (%)	52.2	61.1	48.4	0.001
Hypertension (%)	81.8	83.3	81.2	0.213
Smoking	19.2	18.5	19.5	0.273
Beta-blockers (%)	85.7	87.0	85.1	0.304
ACE/ARB (%)	75.8	74.1	75.8	0.625
Statins (%)	61.5	64.8	60.23	0.236
LVMI (g/m^2^)	146.1 ± 41.23	162.9 ± 38.68	136.1 ± 40.83	<0.001
LVEF (%)	56.90 ± 6.31	52.32 ± 6.18	59.23 ± 5.96	<0.001
Hemoglobin (g/dL)	11.29 ± 1.10	11.91 ± 1.09	10.96 ± 1.02	0.108
Total cholesterol (mg/dL)	189.5 ± 38.64	187.6 ± 37.18	190.3 ± 37.10	0.312
LDL cholesterol (mg/dL)	117.8 ± 31.14	114.6 ± 30.46	118.5 ± 28.75	0.411
HDL cholesterol (mg/dL)	43.99 ± 18.03	44.23 ± 17.55	43.71 ± 15.03	0.699
Triglycerides (mg/dL)	171.1 ± 60.84	168.8 ± 58.77	172.1 ± 53.6	0.328
PTH, range (pg/mL)	380 (0.0-1212)	351 (0.0-899)	440 (0.0-1212)	0.242
Albumin (g/dL)	3.66 ± 0.37	3.68 ± 0.36	3.65 ± 0.32	0.731
CRP, range (mg/dL)	7.34 (0.19-112.1)	10.31 (0.019-112.1)	6.89 (0.28-59.8)	0.021
Troponin T, range (*μ*g/L)	0.057 (0.00-0.773)	0.082 (0.00-0.773)	0.039 (0.029-0.742)	0.069
NT-proBNP (fmol/mL)	321.2 ± 104.5	340.7 ± 104.9	216.8 ± 109.8	0.213
Sodium (mmol/L)	137.6 ± 2.62	137.2 ± 2.61	137.8 ± 2.69	0.434
Potassium (mmol/L)	5.75 ± 0.69	5.76 ± 0.67	5.75 ± 0.63	0.796
Calcium (mmol/L)	2.45 ± 0.23	2.45 ± 0.23	2.46 ± 0.24	0.682
Phosphate (mmol/L)	2.24 ± 0.35	2.20 ± 0.21	2.25 ± 0.23	0.267
Ca x P product (mg^2^/dl^2^)	48.87 ± 9.55	47.19 ± 9.13	49.27 ± 9.51	0.245
TAC (*μ*mol/L)	257.3 ± 31.91	249.2 ± 31.72	260.6 ± 32.84	0.018

CAD: coronary artery disease; MI: history of myocardial infarction; ACE/ARB: angiotensin-converting enzyme inhibitors/angiotensin receptor blockers; LVMI: left ventricular mass index; LVH: left ventricular hypertrophy; LVEF: left ventricle ejection fraction; PTH: parathormone; CRP: C-reactive protein; NT-proBNP: N-terminal prohormone brain natriuretic peptide; TAC: total antioxidant capacity; TOC: total oxidant capacity; oxLDL: oxidized LDL; HSP27: heat shock protein 27.

**Table 2:** Factors influencing QRS-T angle estimated by multivariate stepwise regression analysis.

Dependent variable	Independent variables	*B*	St. error	Beta	*p*
QRS-T	HSP27	- 0.489	0.019	0.336	<0.001
	LVMI	10.35	5.47	0.219	0.009
	Model (*R* = 0.649, *R*^2^ = 0.399)				

HSP27: heat shock protein 27; LVMI: left ventricular mass index.

**Table 3:** Receiver operating characteristic (ROC) curve (Figure 2) of heat shock protein 27 (HSP27) in predicting abnormal QRS-T angle.

	Area	95% confidence interval	Sens/spec (%)	Cut-off
HSP27	0.643	0.621-0.698	0.624/0.601	21.2

## Data Availability

All data used and/or analyzed in the present study are presented in the manuscript or available from the corresponding author on request.
